# No association of complement mannose-binding lectin deficiency with cardiovascular disease in patients with Systemic Lupus Erythematosus

**DOI:** 10.1038/s41598-020-60523-3

**Published:** 2020-02-28

**Authors:** A. Kieninger-Gräfitsch, S. Vogt, C. Ribi, D. Dubler, C. Chizzolini, U. Huynh-Do, M. Osthoff, M. Trendelenburg

**Affiliations:** 1 Division of Internal Medicine and Clinical Immunology Lab, Department of Biomedicine, University Hospital and University, Basel, Switzerland; 2 Department of Immunology and Allergy, University Hospital, Lausanne, Switzerland; 3 Department of Internal Medicine Specialties, Clinical Immunology and Allergy, University Hospital and School of Medicine, Geneva, Switzerland; 4 Department of Nephrology and Hypertension, University Hospital, Bern, Switzerland

**Keywords:** Predictive markers, Biomarkers, Systemic lupus erythematosus

## Abstract

Cardiovascular (CV) morbidity is the major cause of death in patients with Systemic Lupus Erythematosus (SLE). Previous studies on mannose-binding lectin (MBL) gene polymorphisms in SLE patients suggest that low levels of complement MBL are associated with cardiovascular disease (CVD). However, as large studies on MBL deficiency based on resulting MBL plasma concentrations are lacking, the aim of our study was to analyze the association of MBL concentrations with CVD in SLE patients. Plasma MBL levels SLE patients included in the Swiss SLE Cohort Study were quantified by ELISA. Five different CV organ manifestations were documented. Of 373 included patients (85.5% female) 62 patients had at least one CV manifestation. Patients with MBL deficiency (levels below 500 ng/ml or 1000 ng/ml) had no significantly increased frequency of CVD (19.4% vs. 15.2%, P = 0.3 or 17.7% vs. 15.7%, P = 0.7). After adjustment for traditional CV risk factors, MBL levels and positive antiphospholipid serology (APL+) a significant association of CVD with age, hypertension, disease duration and APL+ was demonstrated. In our study of a large cohort of patients with SLE, we could not confirm previous studies suggesting MBL deficiency to be associated with an increased risk for CVD.

## Introduction

Systemic lupus erythematosus (SLE) is an idiopathic, chronic and highly heterogeneous inflammatory disorder, driven by an immune response against self-antigens. The prevalence generally ranges from 20 to 70 per 100.000^1^ and affects both sexes of any age but predominates in women of childbearing age^2^ with a male-to-female ratio of about 9:1^3^.

The etiology of SLE is multifactorial and the complexity of different factors (genetic, hormonal, immunological and environmental) matches the diversity of clinical manifestations in SLE patients^4^. Associated with the presence of immune complexes, ongoing complement system activation leads to inflammation and consumption of complement proteins^5^. This chronic inflammatory state predisposes SLE patients to premature cardiovascular disease (CVD) and infections.

Premature CVD, mostly related to accelerated atherosclerosis^6^, is acknowledged as the major cause of death in SLE patients, regardless of the time after diagnosis^7,8^, resulting in a bimodal mortality curve, first described by Urowitz *et al*. in the 1970s^9^. The 5-year survival after diagnosis nowadays exceeds 90%^10–12^, but this survival rate has not improved since the 1980s.

The prevalence of ischemic heart disease in SLE patients is estimated to be between 3.8 and 16%^13–18^, a 10-fold higher prevalence compared to the general population^19^. Moreover, the risk of myocardial infarction in young women with SLE has been found to be 50 times higher compared to women of similar age^20^ and the risk of stroke was found to be increased by 2–8 fold^19,21,22^.

As traditional Framingham risk factors (smoking, dyslipidemia, diabetes mellitus (DM), hypertension and overweight) cannot fully explain the high rates of ischemic events, SLE is nowadays considered an independent risk factor for CVD^23^.

Several studies suggest that components of the innate immune system, which includes pattern-recognition molecules of the complement system such as mannose-binding lectin (MBL), may play an additional role in the pathogenesis of atherosclerosis and CVD^24–26^. Among those, MBL is a liver-derived serum protein that binds certain sugars on the surface of pathogenic micro-organisms and cellular debris and, as it cannot directly opsonize pathogens, triggers complement activation via the lectin pathway^27^. MBL deficiency (<1000 ng/ml) is mostly caused by a three-point mutation (O alleles) in exon 1 of the MBL gene (in codon 52, 54, 57) that disrupts the assembly of the oligomers and also by a promotor polymorphism (LX) that is associated with low MBL production. However, despite carrying the same genotype, actual MBL levels can vary significantly between individuals^27–29^.

Previous studies indicate that genetic variability in MBL may be involved in the pathogenesis of SLE^30^, more precisely that functional MBL deficiency is associated with an increased susceptibility to SLE^31^. Furthermore, Panda *et al*. described that patients with low MBL-producing genotype have a predisposition to develop SLE^32^. However, data remains controversial as indicated by Losada Lopez *et al*. who only described a tendency to a higher allele B incidence in SLE patients^33^.

Independent of its association with SLE, MBL deficiency has been reported to be associated with an increased risk of atherosclerosis^26^ and coronary artery disease^24^ in the general population, and therefore, to be mostly disadvantageous in this regard^24–26,34^. Of note, Limnell *et al*. even described that MBL deficiency might lead to venous bypass graft occlusion in patients with coronary heart disease^35^. This observation is supported by another report on high MBL levels having a cardioprotective effect, i.e. being associated with a decreased risk for myocardial infarction (MCI) among patients with diabetes^36^. However, data are controversial as some studies show an association of cardiovascular (CV) manifestations with MBL deficiency in the general population but others found no such link^37–39^. This controversy has also recently been summarized by Larsen *et al*.^40^. In addition, an association of deficiency of complement MBL with reduced mortality in patients with MCI undergoing percutaneous coronary intervention^41^ as well as with smaller cerebral infarcts and a favorable outcome^42^ has been described previously. These studies suggest that MBL deficiency – being rather disadvantageous with regard to atherosclerosis and the occurrence of CVD - has an advantageous effect on the outcome after CV events.

Inconsistencies among study results might in part be due to the retrospective character, the relatively small study populations or the focus on MBL2 gene polymorphisms but not on actual MBL plasma levels, which would implicate the MBL deficiency and which are also influenced by non-genetic factors such as inflammation^43^ and thyroid function^44^. Thus, as studies on MBL deficiency based on resulting MBL plasma concentrations were lacking, this study was undertaken to determine the potential association between low MBL levels and the risk for CV events in SLE.

## Methods

### Patients

373 SLE patients included in the Swiss Systemic Lupus Erythematosus Cohort Study (SSCS)^45^ were included in this study. SSCS is a nationwide, multicenter and ethics committee-approved longitudinal study of SLE patients living in Switzerland, conducted in 11 different institutions in 9 Swiss cities. All participants had to fulfill at least 3 out of 11 American College of Rheumatology (ACR) criteria for the classification as SLE at the time of inclusion and had to give written informed consent. In SSCS, the Systemic Lupus Erythematosus Disease Activity (SLEDAI) score with the Safety of Estrogens in Lupus Erythematosus National Assessment (SELENA) modification and Physician’s Global Assessment (PGA) keeps record of the disease activity, while the Systemic Lupus International Collaborating Clinics (SLICC) damage index is used to document chronic organic damage that occurred after diagnosis.

For the analysis of potential confounders, we included common disease activity scores (SELENA-SLEDAI and PGA) and CV risk factors (smoking status, diabetes mellitus (DM), hypercholesterolemia, arterial hypertension, overweight, and positive antiphospholipid serology (APL+)). Since data on packyears (PY) in some cases were missing we additionally compared ever-smokers with non-smokers. We assumed hypercholesterolemia if patients had any form of treated hyperlipidemia, either familial or acquired, and/or met the common laboratory analytic criteria (cholesterol > 5.2 mmol/l and/or low-density lipoprotein (LDL) > 3.4 mmol/l). Similarly, patients were classified as being hypertensive if any treatment with antihypertensive medication was documented. Overweight was classified using the body-mass-index (BMI) that was calculated using the information that was available at study visits but did not take weight changes over time into account. APL+ was defined based on the specification of the SSCS^45^, more specifically as positive finding of antiphospholipid antibody based on an abnormal serum level of IgG or IgM anticardiolipin antibodies or IgG or IgM beta 2-glycoprotein antibodies or a positive test result for lupus anticoagulant using a standard method.

This study was approved by the Ethical Committee of the Canton Basel, Switzerland (Ref. No. EK 262/06), all further involved ethical committees and Swissethics (Ethical Committee of the Canton Vaud, Switzerland Ref No 2017-01434). All subjects gave written informed consent in accordance with the Declaration of Helsinki.

### Evaluation and ascertainment of inclusion criteria and endpoint

Patients solely were selected based on the availability of complete disease activity score (SELENA-SLEDAI, PGA), SLICC damage index, and available plasma sample at the time of study visit. Demographic (e.g. age, ethnic background, disease, and follow-up duration), clinical, laboratory and treatment information were extracted from the cohort database.

Patients were defined as having CVD if at least one CVD of the SLICC index was present. The following arterial thrombotic events were included: (a.) cerebrovascular ischemia (CVI), (b.) coronary heart disease (CHD), (c.) myocardial infarction (MCI), (d.) peripheral artery disease (PAD) and (e.) mesenteric insufficiency.

### Determination of MBL levels and definition of cut-offs

Plasma samples from the 373 SLE patients were collected between 2008 and 2018, which had been exclusively stored at −70 °C and had never been thawed before use. MBL levels were quantified using a mannan-binding enzyme-linked immunosorbent assay (ELISA), as described previously^46^. This assay measures the ability of MBL to bind to a mannan-coated surface. Microtitre plates (Maxi sorp, Thermo Denmark) were coated with 10 µg/ml of mannan (M7504, Sigma-Aldrich USA) in fresh carbonate–bicarbonate buffer (C3041, Sigma-Aldrich USA), pH 9.6, and incubated overnight at 4 °C. Plasma samples were tested as in a sandwich ELISA but with Tris-buffered saline with 0.05% Tween-20 (TBST) (P5927, Sigma-Aldrich USA) and Calciumchlorid-dihydrate (CaCl_2_) (21101, Sigma-Aldrich USA), pH 7.5, as both the diluent and wash buffer. All incubations were carried out at 22 °C. First, the microtitre plates were incubated with samples at a 1:100 dilution for 90 minutes, followed by a biotinylated monoclonal anti-MBL antibody (HYB 131-01B, BioPorto Diagnostics, Denmark) for again 90 minutes to detect the bound MBL. After incubation with ExtrAvidin peroxidase conjugate (E2886, Sigma-Aldrich USA) for 40 minutes the plates were developed with BD TMB substrate solutions (555214, BD USA), stopped after 10 minutes with 0.5 M sulfuric acid (H_2_SO_4)_ (1.00731, Merck Germany) and read immediately at 450 nm on a microplate biokinetics reader (BioTEK, Instruments, USA). MBL levels were calculated against a standard serum pool (ser101, BioPorto Diagnostics Denmark).

Taking the total number of CV events into account, we predefined two different cut-off levels for MBL deficiency, at 500 ng/ml^47^ and 1000 ng/ml^48^, respectively, as described previously.

As a control, measurement of MBL level was repeated in 120 of our 373 included patients using samples from different study visits and serum samples instead of plasma samples to investigate the long-term stability and intra-individual variability of MBL levels in our patients and the potential influence of biosampling on our study results.

### Statistical analysis

First, we examined the baseline characteristics of patients that developed or did not develop CVD using standard descriptive statistical tests. Data are presented as medians (interquartile range (IQR)) or frequencies with percentages when suitable. Beyond the use of predefined cut-offs for the definition of MBL deficiency, a ROC curve was established to explore the data for a potential cutoff level.

Analyzing the long-term stability and intra-individual variability of MBL levels we used Wilcoxon Signed-Ranks Test and Spearman rank correlation for the comparison of the two MBL concentrations that were measured in the same patients at two time points.

Pearson’s qui^2^  test and Fisher’s exact test, respectively, dependent on the group size, were used when comparing the occurrence of CVD and categorical variables. We used the Mann-Whitney U Test and Kruskal-Wallis Test, respectively, when comparing continuous with categorical variables as well as linear regression when comparing two continuous variables. The frequencies of CVD dependent on MBL cut-off levels and MBL levels itself were compared for disease activity index and common CV risk factors as well as APL+. Subsequently, we repeated the analysis for all CVD subcategory.

Last, we analyzed the association of possible risk factors with the presence of CVD using logistic regression models. A multiple logistic regression analysis was performed, taking the dichotomized (yes/no) CVD in total as the dependent variable and those as independent variables that reached statistical significance in the univariate analysis and all variables, corresponding to traditional CV risk factors.

Two-tailed tests and a 5% significance level were used in all analyses. All reported P values of less than 0.05 were considered to indicate statistical significance. The analysis was performed with the statistical package IBM SPSS Statistics 25 for MAC OS X, graphs were performed using the publicly available statistical program R.

In a first exploratory analysis of 177 SLE patients, a strong trend towards a significant difference in plasma MBL concentrations between patients with and without CVD was observed. We calculated an adequately powered study (80%) to require at least 362 SLE patients (alpha = 0.05 incidence of CVD 15% standard deviations as observed in the first 177 patients) in order to be able to detect a difference in MBL plasma levels of at least 450 ng/ml in patients with versus without CVD. Based on this calculation, we selected a SLE patient population of 373 patients to account for potentially missing data.

### Statement

The abstract of this article is present on a university repository website and can be accessed on https://www.sgaim.ch/fileadmin/user_upload/Adaptionen/Congress/BilderHighres/Bilder_FK_2019/Abstracts_2019.pdf. This article is not published nor is under publication elsewhere.

### Compliance with ethical standards

This study was approved by the Ethical Committee of the Canton Basel, Switzerland (Ref No EK 262/06), all further involved ethical committees and Swissethics (Ethical Committee of the Canton Vaud, Switzerland Ref. No. 2017-01434). All procedures performed in this study involving human participants were in accordance with the ethical standards of the research committee and with the 1964 Helsiniki declaration and its later amendments or comparable ethical standards. Informed consent was obtained from all individual participants included in the study.

## Results

### Patients characteristics

The study population comprised 373 patients of whom 319 (85.5%) were female and 54 (14.5%) male. The median (Interquartile Range (IQR)) age at time of first clinical manifestation of SLE was 31.0 (21.6–43.9) years, the median age at the time of blood sampling 43.1 (32.2–54.3) years, the median age at SLICC index assessment 44.8 (34.6–57.5) years, and the median SLE duration at the time of damage assessment since first clinical manifestation was 9.5 (5.2–18.1) years. Of all patients 74.5% were Caucasian, 10% African, 9.7% Asian, 5.1% Native Americans and 0,3% Pacific Islanders. At the time of the study visit 44.2% had a SLEDAI score of 6 or more (median (IQR) SLEDAI score 4 (2–9)) and 53.4% had a PGA equal to 1 or higher.

The basic demographic and clinical characteristics of all patients are summarized in Table 1.

**Table 1:** Demographic characteristics of 373 patients with systemic lupus erythematosus.

	Study Population
Female, n (%)	319/373 (85.5)
**Disease Classification at time of inclusion**	
American College of Rheumatology criteria, median (IQR)	5 (4–6)
**Ethnicity**	
Caucasian, n (%)	278/371 (74.5)
African, n (%)	37/371 (10)
Asian, n (%)	36/371 (9.7)
Pacific Islander, n (%)	1/371 (0.3)
Native American, n (%)	19/371 (5.1)
**Age**	
at first manifestation of SLE, median (IQR), n = 333/373 (89.3%)	31.0 (21.6–43.9)
at blood sampling, median (IQR)	43.1 (32.2–54.3)
at damage assessment, median (IQR)	44.8 (34.6–57.5)
**Disease duration since first manifestation of SLE**	
at blood sampling, median (IQR), n = 333/373 (89.3%)	6.6 (2.3–16.2)
at damage assessment, median (IQR), n = 333/373 (89.3%)	9.5 (5.2–18.1)
**Disease Activity at blood sampling**	
Systemic Lupus Erythematosus Disease Activity Index, ≥6, n (%)	165/373 (44.2)
Physician’s Global Assessment, ≥1, n (%)	199/373 (53.4)
**SLICC Index** at damage assessment, median (IQR), n = 364/373 (97.6%)	2.25 (0–5.7)
**MBL Level** (ng/ml), median (IQR)	1131 (336–2344)
<500 ng/ml, n (%)	129/373 (34.6)
<1000 ng/ml, n (%)	175/373 (46.9)
**Potential confounders**	
Diabetes mellitus, n (%)	25/373 (6.7)
Hypertension, n (%)	127/306 (41.5)
Hypercholesterolemia, n (%)	67/128 (52.3)
Nicotine: Ever-smoker, n (%)	145/342 (42.4)
Body-mass index (kg/m^2^), median (IQR), n = 318/373 (85.3%)	23.4 (20.7–26.6)
Overweight (BMI > 25 kg/m^2^), n (%)	112/318 (35.2)
Positive Antiphospholipid Serology, n (%)	162/371 (43.7)

n = 373, unless otherwise stated, IQR = interquartile range, % = percentage, SLICC = Systemic Lupus International Collaborating Clinics damage assessment.

### MBL - Baseline associations and long-term stability

The median (IQR) MBL level was 1131 (335–2344) ng/ml. 129 patients (34.6%) showed levels below 500 ng/ml and 175 patients (46.9%) below 1000 ng/ml (Table 1). MBL levels had no association with active disease (i.e. SLEDAI ≥ 6 or PGA ≥ 1) at the time of the study visit (p = 0.5 and p = 0.2, respectively). In addition, long-term stability and lack of significant intra-individual variability of MBL levels could be shown in 120 patients (32.2%) whose samples from different study visits were measured twice in different specimens (serum and plasma), at different time points (2014 and 2018) and by two different investigators.

**Figure 1:**
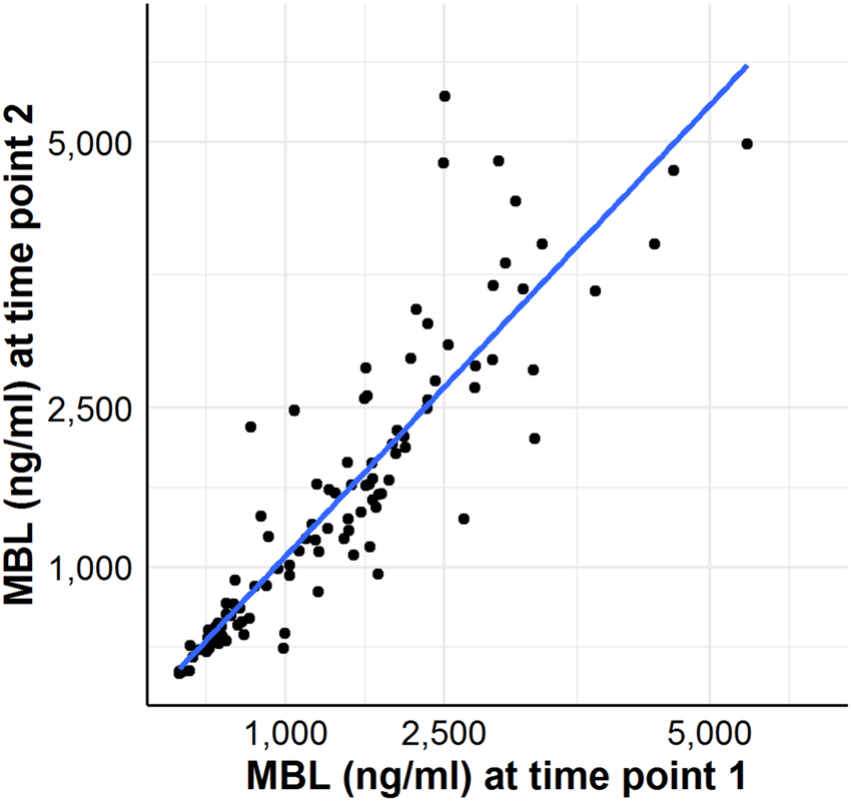
Correlation of MBL levels measured in 120 individuals at two different time points (2014 and 2018). MBL levels between different samples from the same patient correlated significantly as determined in the Spearman Rank Correlation (Correlation Coefficient 0.946, p < 0.001 (2-tailed)).

MBL levels at the two different time points correlated significantly (Spearman’s rho Correlation Coefficient 0.946, p < 0.001, Fig. 1) and did not change over time (median (IQR) levels (1222 (372–2035) ng/ml vs. 1131 (336–2344) ng/ml) as determined by the Wilcoxon Signed Rank Test (Z = −1.717, p = 0.086).

Distribution data of MBL deficiency from healthy Swiss controls were not available. However, likewise to our study population, the frequency of MBL deficiency below 500 ng/ml in SLE patients of our study cohort (median (IQR) 1130 (340–2340) ng/ml, 34.6%) was similar (p = 0.22) to the frequency observed in Swiss scleroderma patients (median (IQR) 1290 (330–2300) ng/ml, 30.3%) as described previously^49^. This study showed a good sample size (=195) and is, therefore, comparable considering the close relationship between systemic sclerosis and SLE.

Furthermore, we investigated the potential association of erythrocyte sedimentation rate (ESR) as marker of inflammation with MBL levels. We observed a very weak correlation between MBL plasma concentration and the corresponding ESR (r = 0.12, p = 0.03) as well as the daily prednisolone (PDN) dose (r = 0.14, 0 = 0.04), respectively.

In addition, using a commonly used pathologic ESR cut-off level of 28 mm/h^50^, we saw a weak association between patients with an elevated ESR and a MBL deficiency below 1000 ng/ml (p = 0.047). However, this association was not found (p = 0.084) when using a 500 ng/ml cut-off. Further, we saw no such association between MBL deficiency and steroid intake, but as expected patients with a higher ESR were more likely to receive higher doses of PDN as part of the treatment (r = 0.3, p < 0.0001).

### CVD

In total, 62 patients (16.6%) had at least one CV manifestation, of these patients 77.4% were Caucasians, 11.3% African, 6.5% Asians and 4.8% Native Americans. 20 patients (5.3%) had more than one CVD manifestation, leading to a total of 93 distinctive CVDs that were documented in the study population. Separated into subcategories, 10.2% of the patients had a CVI (38/373), 7.8% a CHD (20/373), 4.3% a MCI (16/373), 2.4% a PAD (9/373) and 1% (1/373) a documented mesenteric insufficiency. (supplemental Table 1)

Distribution of CVD risk factors in the study population and distribution of CVD according to age, disease duration, ethnicity, CV risk factors, and other confounders are shown in Tables 1 and 2, respectively.

**Table 2:** Distribution of SLE patients with and without cardiovascular disease according to confounders.

	Non-CVD n = 311 (83.4%)	CVD n = 62 (16.6%)	p-value
Age (in years), median (IQR)	41.9 (33.5–53.3)	57.5 (46.0–67.7)	<0.001*
Disease Duration (in years), median (IQR)	8.9 (5.1–16.0)	20.0 (7.0–30.3)	<0.001*
Disease Activity			
SLEDAI ≥ 6, n (%)	138/311 (44.4)	27/62 (43.5)	1.0
PGA ≥ 1, n (%)	168/311 (54.0)	31/62 (50.0)	0.6
SLICC, median (IQR)	1.8 (0–5.1)	2.8 (1.0–6.5)	0.5
Gender			0.047*
Female, n (%)	271/311 (87.1)	48/62 (77.4)	
Male, n (%)	40/311 (12.9)	14/62 (22.6)	
Ethnic background			0.9
MBL Level (in ng/ml), median (IQR)	1180 (339–2344)	955 (306–2389)	0.5
<500 ng/ml, n (%)	104/311 (33.4)	25/62 (40.3)	0.3
<1000 ng/ml, n (%)	144/311 (46.3)	31/62 (50.0)	0.6
Diabetes mellitus, n (%)	18/311 (5.8)	7/62 (11.3)	0.1
Hypertension, n (%)	89/253 (35.2)	38/53 (71.7)	<0.001*
Hypercholesterolemia, n (%)	44/103 (42.7)	23/25 (92)	<0.001*
Nicotine: Ever-smoker	113/286 (39.5)	32/56 (57.1)	0.02*
Pack-years, median (IQR), n = 326/373 (87.4%)	0 (0–5)	7.3 (0–20.0)	<0.001*
BMI (in kg/m^2^), median (IQR)	23.5 (20.6–26.6)	23.4 (21.0–26.8)	0.9
Positive Antiphospholipid Serology, n (%)	124/310 (40.0)	38/61 (62.3)	<0.001*

n = 373, unless otherwise stated, * = statistical significant, IQR = interquartile range, % = percentage, SLE = Systemic Lupus Erythematosus, MBL = Mannose-binding Lectin, SLEDAI = Systemic Lupus Erythematosus Disease Activity Index, PGA = Physician’s Global Assessment, SLICC = Systemic Lupus International Collaborating Clinics damage assessment.

#### *Association of MBL deficiency cut-off levels and continuous MBL levels with CVD and confounders*

Patients with MBL deficiency being defined as plasma concentrations below 500 ng/ml had no significantly increased, nor decreased, frequency of CVD (19.4% vs. 15.2%, P = 0.3). Similarly, MBL levels below 1000 ng/ml were not associated with an increased rate of CVD (17.7% vs. 15.7%, P = 0.6). In addition, MBL deficiency was not associated with the occurrence of any subcategory of CVD.

**Figure 2:**
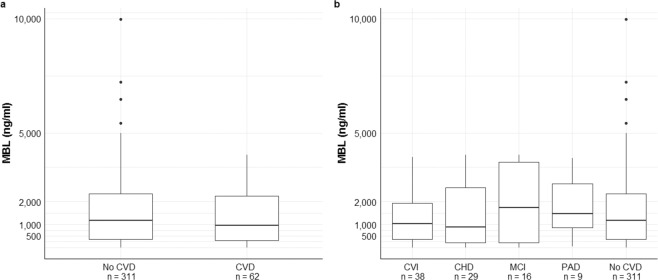
Distribution of MBL levels in 373 SLE patients (**a**) with at least one cardiovascular disease (CVD) and (**b**) with cardiovascular disease according to the subcategory. The horizontal lines depict the median values. (CVD - cardiovascular disease, CVI - cerebrovascular ischemia, CHD - coronary heart disease, MCI - myocardial infarction, PAD - peripheral artery disease).

**Figure 3:**
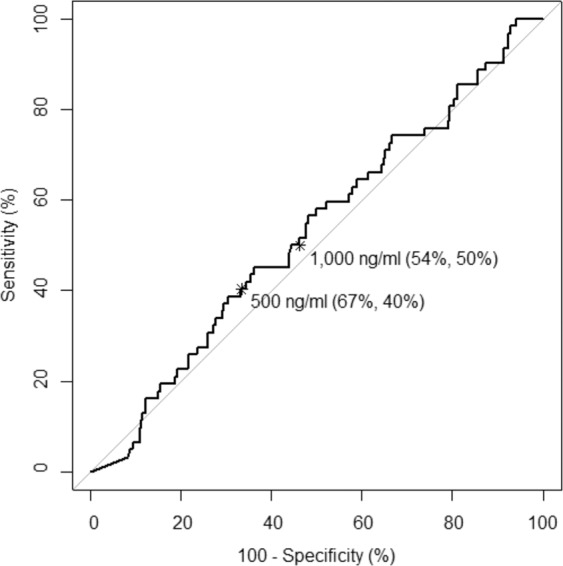
ROC analysis of MBL levels in the prediction of cardiovascular events in SLE patients. The dots represent empirical values of true/false fractions for MBL levels of 0–10,000 ng/ml in increments of CVD. The test characteristics.

Furthermore, MBL levels were similarly distributed in CVD versus no-CVD patients (median (IQR) 1180 (339–2344) ng/ml vs. 955 (306–2389) ng/ml, p = 0.5, Fig. 2a). Last, an explorative receiver operating characteristic (ROC) analysis of MBL levels predicting CVD in SLE patients was performed, but could not identify a candidate cut-off level (area under the curve (AUC) 0.529, Fig. 3).

Similarly, the distribution of MBL levels in subgroup analyses (Supplemental Table 1 and Fig. 2b) did not differ significantly. Of note, mesenteric insufficiency was recorded only once in the whole study population.

Furthermore, our analyses showed that MBL, neither below the deficiency cut-offs nor as a continuous parameter, does not associate with the included confounders including disease duration (data not shown). Likewise, ethnicity was not associated with the occurrence of CVD.

#### *Association of CVD with risk factors and confounders*

With regard to common CVD risk factors, more than half of the patients (52.3%) had hypercholesterolemia, 41.5% had arterial hypertension, 6,7% were diabetic, more than one-third (35.2%) were overweight with a BMI ≥ 25 kg/m^2^ and 42.4% had at least smoked once in their life. In addition, 43.7% of our patients had APL+.

**Figure 4:**
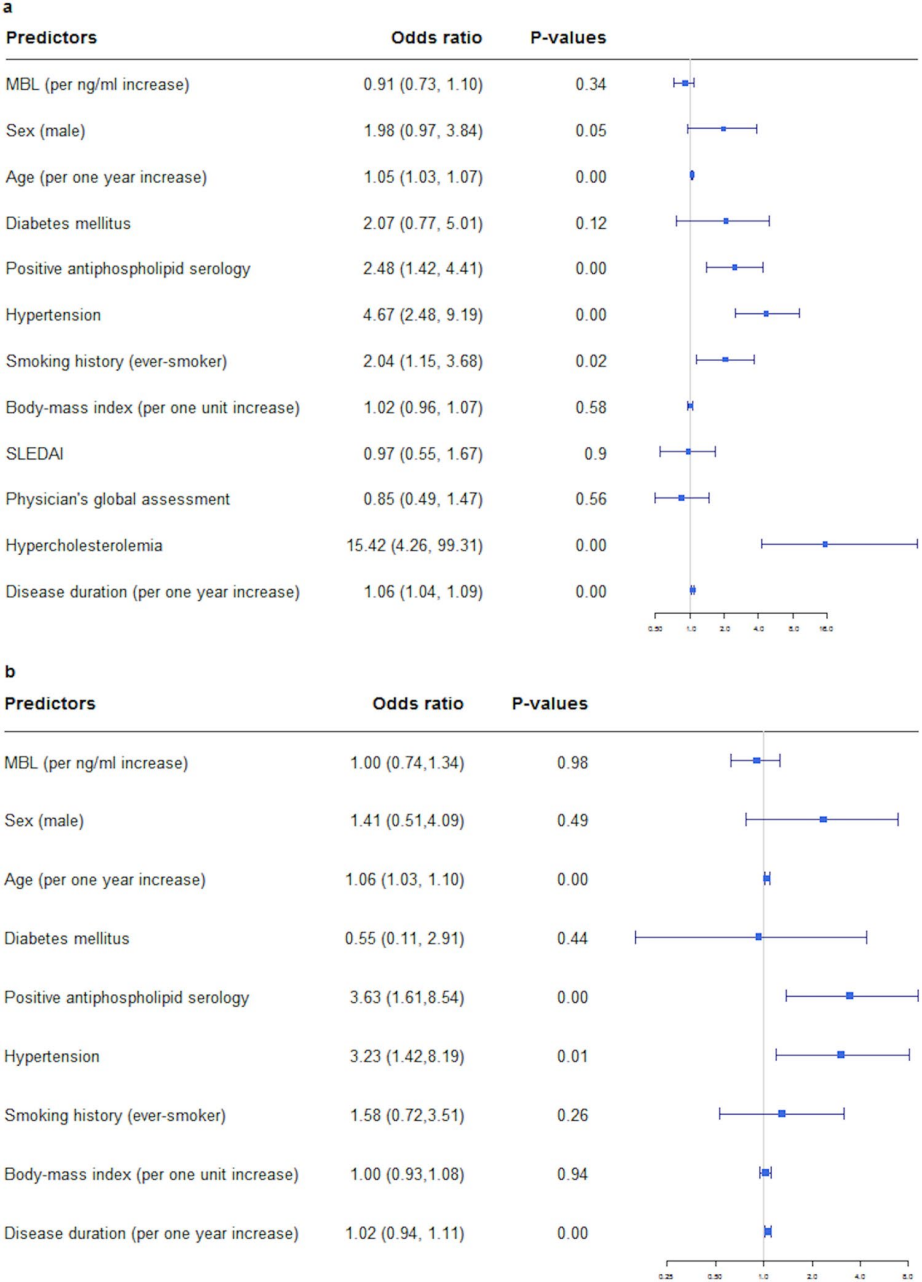
Risk of CVD adjusted for common CV risk factors, APS and MBL levels by univariate and multivariate regression analysis. (**a**) The univariate logistic regression model was performed for MBL levels, sex, age, hypertension, smoking, hypercholesterolemia, BMI, disease activity scores as well as APS. (**b**) We included significant predictors into the multivariate analysis. Due to missing data, hypercholesterolemia had to be excluded to gain explanatory power.

As shown in Table 2, disease duration was longer in patients who had CVD than in those without (median 20.0 vs 8.9 years, p < 0.001). In addition, patients with CVD were older (median age 57.5 vs. 41.9 years, p < 0.001), more likely to be men (22.6% vs. 12.9%, p = 0.047), more frequently smokers (57.1% vs. 39.5%, p = 0.015), more likely to have comorbidities associated with CVD (hypertension 71.7% vs. 35.2%, p < 0.001, hypercholesteremia 92% vs. 42.7, p < 0.001, p = 0.1, APL+ 62.3% vs. 40.0%, p < 0.001, DM 11.3% vs. 5.8%) (Fig. 4) and accrued more damage overall (median SLICC index 2.8 vs. 1.8, p = 0.5). The same results in terms of significance pertained to the CVD subcategories.

The ESR median in patients with CVD was 18.5 mm/h vs. 16.0 mm/h in patients without CVD (p = 0.3). ESR or the dose of PDN were not associated with the incidence of cardiovascular events in the present study (data not shown).

#### *Risk stratification of CVD with potential confounders*

Adjusting the risk of CVD for MBL levels, potential and traditional CV risk factors, revealed a significant association of CVD with male sex (p = 0.05), age (p < 0.001), disease duration (p < 0.001), APL+ (p < 0.001), hypertension (p < 0.001), hypercholesterolemia (p < 0.001) and history of smoking (p = 0.02) in the univariate analysis, while the association with MBL levels p = 0.34), disease activity (SLEDAI p = 0.9, PGA p = 0.56), BMI (p = 0.58) and DM (p = 0.12) did not reach statistical significance. The final multiple logistic regression model including significant variables in the univariate analysis (with the exception of hypercholesterolemia due to missing values in two third of the patients) revealed an independent significant association between CVD in SLE patients with age (p < 0.001), disease duration (p < 0.001), hypertension (p < 0.005) and APL+ (p < 0.003), while the association with male sex (p = 0.49) and smoking (p = 0.26) did not reach significance anymore (Fig. 4).

## Discussion

Compared to the general population^20^ patients with SLE have an increased risk for CV morbidity and preclinical atherosclerosis^51,52^. Traditional CV risk factors cannot fully explain this observation^19^, suggesting that SLE disease-specific factors may play an additional role^53^. Previous studies described deficiency of complement MBL to be related to CVD in the general population^36^ and in SLE patients^54,55^.

With a focus on patients with SLE, previous studies on MBL gene polymorphisms suggest that low-producing MBL genotypes are associated with increased intima-media thickness^56^ and arterial thrombosis, more specifically with coronary events^55^. This is in accordance with another study in which MBL-deficient SLE patients were found to have a 3.3 fold increased risk of CVD^54^.

However, Calvo-Alen *et al*. found an association of MBL deficiency with cerebrovascular incidents (CVI) but not with ischemic heart disease^57^, and Jönsen *et al*. could show that classical risk factors (smoking, hypertension, low alcohol intake, elevated triglyceride concentration) were relatively more important for the development of CVD than MBL deficiency^58^.

Our study was designed to elucidate whether MBL deficiency, based on the resulting blood concentrations, is also associated with an increased incidence of CVD in SLE patients. To address this question, we measured MBL plasma levels and investigated their potential association with clinical CV manifestation and with the main CV risk factors.

Our results suggest that MBL deficiency is not a determinant of CVD in SLE patients, independent of other risk factors.

In part, our findings are in contrast to data reported by previous studies. These differences can be explained by a number of factors. First, our study focused on the determination of plasma MBL concentrations and not the genotypes^54,55,57,58^. With regard to the variability of MBL levels within one genotype^27,29^, MBL plasma levels seem to be more discriminative in terms of immunological functionality^36^. Second, we focused on all CVD out of the 12 organ systems that are recorded by the SLICC damage index, including CVI, CHD, MCI PAD and mesenteric insufficiency. Garred *et al*.^59^ were the first to describe a higher frequency of thrombotic disease in SLE patients with MBL low-producing genotypes and this analysis was limited to arterial thrombosis in a later study by ∅hlenschlaeger *et al*.^55^, while Calvo-Alen *et al*.^57^ restricted their analysis to CVI. Of interest, Jönsen *et al*.^58^ and Calvo-Alen *et al*.^57^ used a similar outcome as in our study, allowing a better direct comparison between the studies. While the latter one showed an association with CVI, the first one, similarly to our study, rather described traditional CV risk factors to be primarily associated with CVD.

In direct comparison with the study by Jönsen *et al*.^58^ our study population had a considerably shorter follow-up time (in years) (median (range) 14 (0–49) vs. median (IQR) 9.5 (5.2–18.1)) but a somewhat higher age (in years) of the patients (median (range) 40 (10–83) vs. median (IQR) 44.8 (34.6–57.5)).

Another parameter that needs to be considered is the limited number of patients and, as a consequence, the limited number of patients with CVD investigated in previous studies. In comparison, our study evaluated a larger study population than the studies by ∅hlenschlaeger *et al*.^55^, Jönsen *et al*.^58^ and Font *et al*.^54^ but slightly less patients than the study by Calvo *et al*.^57^ Although in our study MBL deficiency was numerically more common in the group of SLE patients with CVD than in the Non-CVD group (40.3% vs. 33.4% below 500 ng/ml and 50.0% vs. 46.3% below 1000 ng/ml), this difference was not significant, which could be due to the relatively small number of events in total. In this context, it is of interest to note that patients in the study by ∅hlenschlaeger *et al*.^55^ developed an arterial thrombosis in 26%, a frequency 10% higher than in our study population. Likewise, Calvo-Alen *et al*.^57^, Jönsen *et al*.^58^ and Font *et al*.^54^ showed a considerably higher rate of CV events, which might be related to the younger age of our SLE patients and the relatively short follow-up time in our study. As a consequence, in spite of the relatively large number of SLE patient, our study might be limited in power due to its comparatively small number of CVD in total.

A further limitation that needs to be mentioned is that the damage assessment was carried out using the SLICC index and not with a specific focus on CVD at the time of data collection. Last, risk stratification analyses could not be adjusted to the thyroid function, but, according to our database records, the vast majority of our patients had no evidence of thyroid disease.

In balance with these limitations, the strength of our study is the large cohort size of SLE patients being prospectively included in the Swiss SLE Cohort Study (SSCS). The study population included both genders in a typical distribution as observed in patients with SLE and was shown to have typical patient characteristics. In addition, we focused on the determination of plasma MBL concentrations which maybe best reflect functionality. MBL levels in our patients were found to be stable over time and not related to SLE disease activity.

In this setting, we could not find a significant association between MBL deficiency and the occurrence of CVD in SLE patients. However, large prospective studies with long follow-ups would be required to definitely exclude a role of MBL in SLE-associated CVD.

## Supplementary information

Supplementary information

## Data Availability

The data generated or analyzed during the current study are available from the corresponding author on reasonable request.
