# Supplementary material for: No association of complement mannose-binding lectin deficiency with cardiovascular disease in patients with Systemic Lupus Erythematosus
Source: Sci Rep. 2020 Feb 28;10:3693. doi: 10.1038/s41598-020-60523-3 (PMC7048794; doi:10.1038/s41598-020-60523-3)
Supplement: Supplementary file 1 — Supplementary information [file 41598_2020_60523_MOESM1_ESM.pdf]

No association of complement mannose-binding lectin deficiency with cardiovascular disease in patients with Systemic Lupus Erythematosus

A.Kieninger-Gräfitsch[1*], S.Vogt[1], C.Ribi[2], D.Dubler[1], C.Chizzolini[3], U.Huynh-Do[4], M.Osthoff[1+], M.Trendelenburg[1+]

Supplement --- Table 1| Demographic characteristics and distribution of cardiovascular disease in total and every subcategory.

	Study Population	*Cardiovascular Disease*	*Cerebrovascular Disease*	*Coronary Heart Disease*	*Myocardial Infarction*	*Peripheral Artery Disease*	*Mesenteric Insufficiency*
Count, n (%)	373	62/373 (16.6)	38/373 (10.2)	29/373 (7.8)	16/373 (4.3)	9/373 (2.4)	1/373 (0.3)
Age, median (IQR)	44.8 (34.6-57.5)	57.7 (45.3-67.7)	56.2 (43.4-64.6)	65.7 (52.4-71.7)	67.6 (49.0-75.0)	59.7 (44.9-76.3)	67.7**
Disease Duration, median (IQR)	9.5 (5.2-18.1)	20.0 (7.0-30.3)	20.0 (7.2-31.0)	21.8 (6.7-31.4)	19.0 (6.6-29.5)	24.9 (10.0-29.5)	35.8**
Disease Activity							
SLEDAI ≥ 6, n (%)	165/373 (44.2)	27/62 (43.5)	16/38 (42.1)	15/29 (51.7)	11/16 (68.8)	3/9 (33.3)	0/1 (0)
PGA ≥ 1, n (%)	199/373 (53.4)	31/62 (50.0)	23/38 (60.5)	13/29 (44.8)	8/16 (50.0)	5/9 (55.6)	0/1 (0)
SLICC, median (IQR)	2.3 (0-5.7)	2.8 (1.0-6.5)	3.0 (1.0-6.5)	2.2 (1.0-6.6)	2.2 (0.9-4.4)	2.7 (1.3-3.7)	8**
Gender							
Female, n (%)	319/373 (85.5)	48/62 (77.4)	31/38 (81.6)	21/29 (72.4)	13/16 (81.3)	8/9 (88.9)	1/1 (100)
Male, n (%)	54/373 (14.5)	14/62 (22.6)	7/38 (18.4)	7/29 (27.6)	3/16 (18.8)	1/9 (11.1)	
Ethnic Background							
Caucasian, n (%)	278/371 (74.5)	48/62 (77.4)	26/38 (68.4)	25/29 (86.2)	13/16 (81.3)	7/9 (77.8)	1/1 (100)
African, n (%)	37/371 (10)	7/62 (11.3)	6/38 (15.8)	2/29 (6.9)	2/16 (12.5)	1/9 (11.1)	
Asian, n (%)	36/371 (9.7)	4/62 (6.5)	3/38 (7.9)	2/29 (6.9)	1/16 (6.3)	0/9 (0)	
Pacific Islander, n (%)	1/371 (0.3)	0/62 (0)	0/38 (0)	0/29 (0)	0/16 (0)	0/9 (0)	
Native American, n (%)	19/371 (5.1)	3/62 (4.8)	3/38 (7.9)	0/29 (0)	0/16 (0)	1/9 (11.1)	
MBL Level (ng/ml), median (IQR)	1131 (336-2344)	955 (306-2389)	1032 (346-1947)	881 (131-2624)	1733 (114-3783)	1470 (586-3347)	0**
< 500 ng/ml, n (%)	129/373 (34.6)	25/62 (40.3)	15/38 (39.5)	12/29 (41.4)	6/16 (37.5)	2/9 (22.2)	1/1 (100)
< 1000 ng/ml, n (%)	175/373 (46.9)	31/62 (50.0)	18/38 (47.4)	16/29 (55.2)	7/16 (43.8)	3/9 (33.3)	
Diabetes mellitus, n (%)	25/373 (6.7)	7/62 (11.3)	5/38 (13.2)	4/29 (13.8)	3/16 (18.8)	1/9 (11.1)	1/1 (100)
Hypertension, n (%)	127/306 (41.5)	38/53 (71.7)	22/31 (71)	22/27 (81.5)	13/15 (86.7)	8/9 (88.9)	1/1 (100)
Hypercholesterolemia, n (%)	67/128 (52.3)	23/25 (92)	12/12 (100)	14/16 (87.5)	8/9 (88.9)	4/5 (80.0)	1/1 (100)
Nicotine: Ever-smoker, n (%)	145/342 (42.4)	32/56 (57.1)	20/36 (55.6)	16/26 (61.5)	11/15 (73.3)	6/9 (66.7)	*
Body-mass index: Overweight (>25), n (%)	112/318 (35.2)	17/52 (32.7)	12/32 (37.5)	7/23 (30.4)	4/15 (26.7)	3/8 (37.5)	1/1 (100)
Positive Antiphospholipid Serology, n (%)	162/371 (43.7)	38/61 (62.3)	29/38 (76.3)	13/28 (46.4)	8/15 (53.3)	7/9 (77.8)	

SLEDAI = Systemic Lupus Erythematosus Disease Activity Index
PGA = Physician`s Global Assessment
SLICC = Systemic Lupus International Collaborating Clinics damage assessment
* missing data for event case, hence not described
** single event, hence exact number and no IQR
